# Diabetes, plasma glucose and incidence of pancreatic cancer: A prospective study of 0.5 million Chinese adults and a meta‐analysis of 22 cohort studies

**DOI:** 10.1002/ijc.30599

**Published:** 2017-02-08

**Authors:** Yuanjie Pang, Christiana Kartsonaki, Yu Guo, Fiona Bragg, Ling Yang, Zheng Bian, Yiping Chen, Andri Iona, Iona Y Millwood, Jun Lv, Canqing Yu, Junshi Chen, Liming Li, Michael V Holmes, Zhengming Chen

**Affiliations:** ^1^ Clinical Trial Service Unit & Epidemiological Studies Unit (CTSU), Nuffield Department of Population HealthUniversity of OxfordOxfordUnited Kingdom; ^2^ Medical Research Council Population Health Research Unit (MRC PHRU) at the University of OxfordOxfordUnited Kingdom; ^3^ Chinese Academy of Medical SciencesBeijing100730China; ^4^ School of Public HealthPeking UniversityBeijing100191China; ^5^ National Center for Food Safety Risk AssessmentBeijing100021China

**Keywords:** diabetes, plasma glucose, pancreatic cancer, Chinese

## Abstract

Diabetes is associated with an increased risk of pancreatic cancer (PC) in Western populations. Uncertainty remains, however, about the relevance of plasma glucose for PC among people without diabetes and about the associations of diabetes and high blood glucose with PC in China where the increase in diabetes prevalence has been very recent. The prospective China Kadoorie Biobank (CKB) study recruited 512,000 adults aged 30‐79 years from 10 diverse areas of China during 2004‐2008, recording 595 PC cases during 8 years of follow‐up. Cox regression yielded adjusted hazard ratios (HRs) for PC associated with diabetes (previously diagnosed or screen‐detected) and, among those without previously diagnosed diabetes, with levels of random plasma glucose (RPG). These were further meta‐analysed with 22 published prospective studies. Overall 5.8% of CKB participants had diabetes at baseline. Diabetes was associated with almost twofold increased risk of PC (adjusted HR = 1.87, 95% CI 1.48‐2.37), with excess risk higher in those with longer duration since diagnosis (*p* for trend = 0.01). Among those without previously diagnosed diabetes, each 1 mmol/L higher usual RPG was associated with a HR of 1.12 (1.04‐1.21). In meta‐analysis of CKB and 22 other studies, previously diagnosed diabetes was associated with a 52% excess risk (1.52, 1.43‐1.63). Among those without diabetes, each 1 mmol/L higher blood glucose was associated with a 15% (1.15, 1.09‐1.21) excess risk. In Chinese and non‐Chinese populations, diabetes and higher blood glucose levels among those without diabetes are associated with an increased risk of PC.

## Abstract 2

**What's new?**

Diabetes is associated with increased risk of pancreatic cancer (PC) in Western populations, but uncertainty remains about the relevance of plasma glucose among people without diabetes. In this first prospective study investigating the association of diabetes with PC risk in China, where the increase in diabetes prevalence has been very recent, diabetes was associated with an almost twofold increased risk of PC, with random plasma glucose being positively associated with PC risk among participants without prior diagnosis of diabetes. In meta‐analysis, the results were consistent with previous evidence in Western populations, highlighting diabetes as a potential aetiological factor of PC.

**Abbreviations**
BMI: body mass index
CI: confidence interval
CKB: China Kadoorie Biobank
DSP: Disease Surveillance Points
FBG: fasting blood glucose
FPG: fasting plasma glucose
HR: hazard ratio
ICD‐10: International Classification of Diseases, 10th Revision
IHD: ischemic heart disease
MET: metabolic equivalent of task
PC: pancreatic cancer
RBG: random blood glucose
RPG: random plasma glucose
RR: relative risk
SBP: systolic blood pressure
SD: standard deviation

## Introduction

Although pancreatic cancer (PC) is ranked as the 12th commonest cancer globally,^1^, ^2^ it has the highest case fatality among all cancers, with a 5‐year survival of only 3–5%,^3^ and the incidence is rising in many countries, including China.^4^ Previous studies of mostly Western populations have reported that individuals with diabetes or pre‐diabetes (fasting plasma glucose 5.6–6.9 mmol/L or post‐load plasma glucose 7.8–11.0 mmol/L^5^) have an increased risk of PC, although the magnitude of the risk in different populations and relevance of diabetes duration is less clear.^6^, ^7^, ^8^ In those without diabetes, uncertainty remains as to whether blood glucose levels are associated with risk of PC, and, if so, whether the association shows a continuous dose–response relationship. Furthermore, most studies have focused on fasting blood glucose (FBG) levels, with little data on random blood glucose (RBG),^9^ which is arguably a more practical and relevant measure because people spend most time in a non‐fasting state.^10^

In recent decades, there has been a substantial increase in diabetes incidence in China,^11^ with a high proportion of diabetes cases undetected.^11^ Furthermore, the increase in diabetes prevalence is so recent that the true impact of diabetes on risk of PC and other diseases may not yet have emerged. Despite this, there is no prospective evidence available in China about the associations of diabetes and blood glucose with risk of PC. The present large prospective China Kadoorie Biobank (CKB) study examines these associations. To help reliably quantify the strength of the associations, we also meta‐analysed CKB results with published evidence.

## Material and Methods

### Study population

Details of the CKB design, survey methods and population characteristics have been described elsewhere.^12^ Briefly, 512,891 participants (210,222 men and 302,669 women) aged 30–79 years were recruited into the study from 10 geographically defined localities (5 urban and 5 rural) in China during 2004–2008. The study areas were selected to provide diversity in risk exposure and disease patterns, while taking into account population stability, quality of mortality and morbidity registries, capacity and long‐term commitment within the areas. Prior international, national and regional ethical approvals were obtained, and all participants provided written informed consent.

### Data collection

At local study assessment clinics, participants completed an interviewer‐administered laptop‐based questionnaire on socio‐demographic characteristics, smoking, alcohol consumption, diet, physical activity, personal and family medical history and current medication. A range of physical measurements were recorded by trained technicians, including height, weight, hip and waist circumference, bio‐impedance, lung function, blood pressure and heart rate, using calibrated instruments with standard protocols.

A 10‐ml non‐fasting (with the time since the participant last ate recorded) blood sample was collected from participants into an EDTA vacutainer (BD Hemogard^TM^, USA). Immediate on‐site testing of random plasma glucose (RPG) level was undertaken using the SureStep Plus System (Johnson & Johnson), regularly calibrated with manufacturer quality control solution. Participants with glucose levels ≥7.8 mmol/L and <11.1 mmol/L were invited to return for a fasting plasma glucose (FPG) test the next day. RPG data were unavailable for 8,306 participants (because of a delay in making the on‐site test available in certain regions).

Previously diagnosed diabetes was defined by the question 'Has a doctor ever told you that you had diabetes?'. Among positive respondents, additional information about age at first diagnosis and current use of certain medications for diabetes (e.g. insulin and metformin) and cardiovascular diseases (e.g. aspirin, lipid and blood pressure lowering agents) was collected. Among those without previously diagnosed diabetes, screen‐detected diabetes was defined as RPG ≥7.0 mmol/L and time since last eating ≥8 h, or ≥11.1 mmol/L with time since last eating <8 h, or a FPG ≥7.0 mmol/L on subsequent testing.^13^ For those with previously diagnosed diabetes, duration of diabetes was defined as the time interval between diagnosis and baseline visit.

From August to October 2008 (∼2.6 years after the baseline survey) a resurvey was done on 19,788 (∼5%) randomly selected surviving participants. The data collection and survey procedures were much the same as in the baseline survey. RPG data were available for 19,712 (99.6%) participants.

### Follow‐up for cancer incidence and mortality

The vital status of each participant was determined periodically through China CDC's Disease Surveillance Points (DSP) system^14^ and national health insurance system, supplemented by regular checks against local residential and administrative records and by annual active confirmation through street committees or village administrators. In addition, information about major diseases and any episodes of hospitalisation was collected through linkage, via each participant's unique national identification number, with disease registries (for cancer, ischemic heart disease [IHD], stroke and diabetes) and national health insurance claims databases. All death or hospitalised events were coded using International Classification of Diseases, 10th Revision (ICD‐10) by trained DSP staff who were blinded to baseline information^12^. By 1.1.2015, 30,582 (6%) participants had died, 3,898 (0.8%) were lost to follow‐up and 21,266 (4.1%) had developed cancer, including 595 (0.1%) with PC (ICD‐10 C25).

### Statistical analyses

This study excluded participants with a prior history of cancer at baseline (*n* = 2,577), leaving 510,314 for the main analysis.

The prevalence and mean values of participants' baseline characteristics by diabetes status at baseline were calculated using direct standardisation to the age (in 5‐year age groups), sex and area structure of the CKB population.

Cox regression models were used to obtain adjusted hazard ratios (HRs) of PC incidence associated with diabetes and RPG levels, stratified by age at risk (5‐year groups), sex and study region (10 regions) and adjusted for education (4 groups), smoking (3 groups: never, occasional, or ever regular) and alcohol (5 groups: abstainers, ex‐weekly drinkers, reduced‐intake drinkers, occasional drinkers or weekly drinkers). Duration of diabetes was categorised into four groups (no diabetes [reference], screen‐detected diabetes, <5 years and ≥5 years). The analysis for RPG was conducted in participants without previously diagnosed diabetes (*n* = 486,189). RPG was categorised into three groups (≤5.5 [reference], >5.5–7.0 and ≥7.0 mmol/L), selected to include the FPG thresholds for impaired fasting glucose and diabetes. RPG was also modeled as a continuous variable to estimate risk associated with a 1 mmol/L increase in RPG. The analysis for RPG was additionally adjusted for fasting time. For analyses involving more than two categories, all HRs are presented with 'floating' standard errors to facilitate comparisons between groups.^15^

To adjust for regression dilution bias, we calculated the correlation between RPG at baseline visit and RPG measured at resurvey among 19,338 participants (Pearson correlation coefficient 0.45 [95% CI 0.44–0.46]). Log HR estimates (and corresponding standard errors) per 1 mmol/L higher plasma glucose were divided by this correlation to obtain regression dilution‐adjusted estimates.^16^ In sensitivity analyses, we excluded PC cases that occurred during the first 2 years and 5 years of follow‐up, separately. We also used Cox models with a time‐updated exposure counting individuals with incident diabetes as exposed from their time of diagnosis. The analyses were done using SAS version 9.3 and R version 2.14.2.

### Meta‐analysis

We followed PRISMA guidelines for conducting a systematic review and meta‐analysis.^17^ PubMed and Embase were searched for studies published in English from database inception to March 2016. The precise search terms are provided in the Supporting Information Data. Inclusion criteria were prospective cohort studies, case–cohort studies, or nested case–control studies (with diabetes ascertained at baseline) reporting the association between diabetes and PC incidence or mortality. Bibliographies of included studies and related reviews were manually searched for additional eligible articles.

We calculated relative risks (RR) for diabetes using fixed effects modeling and fitted random effects models as a sensitivity analysis (Supporting Information Table 1). The main subgroup analyses included study design, geographical region, diagnostic method (previously diagnosed, screen‐detected or both), different adjustment in the statistical models, whether or not studies excluded initial years of follow‐up and duration of diabetes. Meta‐regression analyses were conducted to assess heterogeneity between subgroups. Heterogeneity between studies was assessed using *I* ^2^ and Cochran's *Q* test. Publication bias was assessed visually by inspecting the funnel plots for asymmetry and tested with Egger's test,^18^ with the result considered to indicate small study effects when *p* <0.05.

As we could not identify any prospective studies on blood glucose and PC that have been published since the most recent systematic review and meta‐analysis on this topic,^8^ we meta‐analysed the results from CKB with the studies included in that meta‐analysis. We excluded studies that measured only HbA1c. We estimated the RR per 1 mmol/L higher blood glucose within the range of normoglycaemia from individual studies by quantifying the study‐specific linear trends between exposure and outcome using the method described by Greenland and Longnecker^19^ (which allows for non‐independence of relative risk estimates within each study). We then pooled the estimated linear trends using inverse‐variance weighted fixed effects meta‐analysis.

## Results

Among the 510,314 participants in CKB, the mean (SD) age was 51.5 (10.7) years, and 59% were women. The prevalence of previously diagnosed and screen‐detected diabetes was 3.1% and 2.7%, respectively. The mean (SD) RBG was 5.7 (1.1), 13.3 (5.4) and 11.8 (5.7) mmol/L among participants without any diabetes, with screen‐detected diabetes and with previously diagnosed diabetes, respectively. Individuals with diabetes were older, and, after adjustment for age, sex and region, more likely to have higher BMI, higher SBP, lower physical activity and to have a family history of diabetes (Table 1). Among those with previously diagnosed diabetes, the median age at first diagnosis was 53 years and the median duration since diagnosis was 6 years.

**Table 1:** Baseline characteristics by diabetes status in CKB

		Diabetes status		
Variable^a^	No diabetes (n=480,307)	Total (n=30,007)	Previously diagnosed (n=16,000)	Screen‐detected (n=14,007)
Age (SD), year	51.1 (10.6)	57.2 (9.6)	58.3 (9.1)	55.9 (9.9)
Female, %	276205 (58.8)	17697 (61.4)	9462 (62.0)	8232 (60.7)
Urban region, %	201987 (43.0)	17525 (60.8)	9936 (65.1)	7568 (55.8)
Middle school and higher, %	231284 (49.2)	13955 (48.4)	7682 (50.3)	6344 (46.8)
Annual household income ≥ 35 000 Yuan, %	84284 (17.9)	5480 (19.0)	3042 (19.9)	2521 (18.6)
Regular smoker, %				
Male	134378 (67.6)	7196 (67.7)	3607 (67.7)	3589 (64.3)
Female	7766 (3.1)	781 (2.8)	458 (2.8)	323 (3.1)
Weekly drinker, %				
Male	66248 (36.0)	3493 (33.6)	1402 (33.6)	2091 (21.8)
Female	5990 (1.9)	232 (2.1)	78 (2.1)	154 (0.7)
Adult BMI (SD), kg/m^2^	23.6 (3.3)	24.9 (3.6)	24.6 (3.5)	25.1 (3.7)
BMI at age 25 (SD), kg/m^2^	21.9 (2.5)	22.8 (3.1)	23.2 (3.2)	22.5 (2.9)
SBP (SD), mmHg	130.6 (21.0)	138.5 (22.6)	137.5 (22.6)	139.3 (22.7)
Random plasma glucose (SD), mmol/L	5.7 (1.1)	12.6 (5.6)	11.8 (5.7)	13.3 (5.4)
Total physical activity (SD), MET h/day	21.3 (13.9)	18.8 (11.9)	17.5 (10.6)	19.8 (12.9)
Family history of diabetes, %	21258 (4.5)	3506 (12.2)	2444 (16.0)	1244 (9.2)
Family history of cancer, %	65431 (13.9)	4103 (14.2)	2313 (15.2)	1817 (13.4)

a Results were adjusted for age, sex and region (where appropriate). Values are presented as number (percentage) for categorical variables and mean (SD) for continuous variable. Abbreviations: BMI = body mass index; SBP = systolic blood pressure; MET = metabolic equivalent of task. 1 mmol/L= 18 mg/dL.

### Diabetes and risk of PC

Individuals with diabetes had an approximate doubling of risk for PC (adjusted HR = 1.87, 95% CI 1.48–2.37, Table 2). The HR was greater for previously diagnosed than for screen‐detected diabetes (2.08 [1.56–2.76] vs. 1.54 [1.08–2.20]). Compared with participants without diabetes, the risk also increased progressively with increasing duration of diabetes, with adjusted HRs of 1.54 (1.07–2.20), 2.11 (1.43–3.09) and 2.16 (1.50–3.11) for those with screen‐detected diabetes, <5 years and ≥5 years of duration, respectively (*p* for trend = 0.01) (Table 2). Non‐floating CIs for diabetes and RPG are shown in Supporting Information Table 2. Additional adjustment for BMI did not alter the results (Supporting Information Table 3). The associations persisted after excluding PC cases that occurred during the first 2 or 5 years of follow‐up (Supporting Information Table 4). The associations were similar when individuals with incident diabetes after baseline were also considered as exposed from their time of diagnosis (Supporting Information Table 2). The associations with PC mortality were similar (Supporting Information Table 5).

**Table 2:** Adjusted HRs for PC by diabetes and random plasma glucose at baseline in CKB

	No. events	No. people	Rate, per 100,000	Person‐time, PYAR	HR (95% CI) a
*Diabetes status*					
No diabetes	499	480,307	103.89	3,894,806	Reference
Total diabetes	86	30,007	286.60	230,790	1.87 (1.48, 2.37)
*Diabetes status*					
No diabetes	499	480,307	103.89	3,894,806	1.00 (0.90, 1.11)
Screen‐detected	30	14,007	214.18	108,552	1.54 (1.08, 2.20)
Previously diagnosed	56	16,000	350.00	122,238	2.08 (1.56, 2.76)
*Duration of diabetes* b					
No diabetes	499	480,307	103.89	3,894,806	1.00 (0.90, 1.11)
Screen‐detected	30	14,007	214.18	108,552	1.54 (1.07, 2.20)
Previously diagnosed <5 years	21	8,341	311.71	64,714	2.11 (1.43, 3.09)
≥5 years	30	7,659	391.70	57,374	2.16 (1.50, 3.11)
*p for trend*					*0.01*
*Random plasma glucose* (mmol/L)^c^					
≤5.5	207	242,737	85.28	1,893,962	1.00 (0.86, 1.16)
>5.5‐7.0	208	172,701	120.44	1,473,516	1.11 (0.97, 1.24)
≥7.0	97	70,751	137.10	555,171	1.22 (1.00, 1.50)
per 1 mmol/L^d^	512	486,189	105.31	3,922,649	1.12 (1.04, 1.21)

a Estimates were stratified by age at risk, sex and region, and adjusted for age at baseline, education, smoking and alcohol.

b Diabetes duration data were missing or implausible for 20 participants.

c The analysis for random plasma glucose was restricted to participants without previously diagnosed diabetes and further adjusted for fasting time.

d HR per 1 mmol/L in blood glucose was corrected for regression dilution. The regression dilution ratio is 0.45. 1 mmol/L= 18 mg/dL.

Abbreviations: PYAR = person years at risk.

### Blood glucose and risk of PC

**Figure 1:**
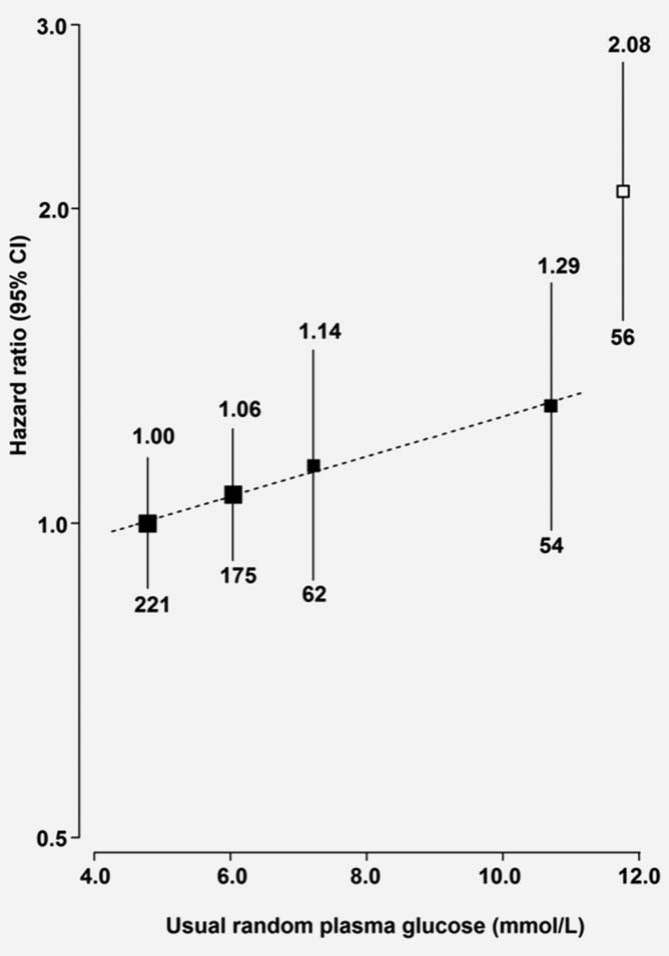
Adjusted HRs for PC by usual random plasma glucose in CKB. Random plasma glucose levels for participants without previously diagnosed diabetes at baseline were classified as ≤5.5 (reference), 5.6‐6.7, 6.8‐7.7 and ≥7.8 mmol/L. Hazard ratios (HRs) were plotted against the mean usual random plasma glucose level in each group. The sizes of the data markers are proportional to the inverse of the variance of the log HRs. The numbers above the 95% CI are point estimates for HRs, and the numbers below are numbers of PC cases for each category. The models were stratified by age at risk, sex and region and adjusted for age at baseline, education, smoking, alcohol and fasting time. Black boxes represent the HRs for random plasma glucose and the open box represents previously diagnosed diabetes.

In participants without previously diagnosed diabetes, there was a positive association of RPG with risk of PC (Table 2). The association was log‐linear, with each 1 mmol/L higher RPG associated with an adjusted HR of 1.12 (1.04–1.21) after adjusting for regression dilution bias (Fig. 1).

### Meta‐analysis of CKB with published studies

Our systematic review of diabetes and PC identified 4,245 eligible studies, of which 33 met the inclusion criteria (Supporting Information Fig. 1). These included 16 prospective cohort studies, four nested case–control studies, one case–cohort study and one pooled analysis of prospective cohort studies, which were included in the meta‐analysis (Supporting Information Data). Of those 22 prospective studies, 16 used self‐reported physician‐diagnosed diabetes (i.e. previously diagnosed), five combined both previously diagnosed and screen‐detected diabetes through specific tests (e.g. FBG, post‐load blood glucose, or HbA1c), and one reported previously diagnosed and screen‐detected diabetes separately (Supporting Information Data). We further identified 11 record linkage studies (Supporting Information Data) but they were not included in the meta‐analysis due to large heterogeneity in study characteristics (Supporting Information Fig. 2). Four studies had information on duration of diabetes (Supporting Information Data). The detailed characteristics of the included studies are provided in Supporting Information Table 6.

**Figure 2:**
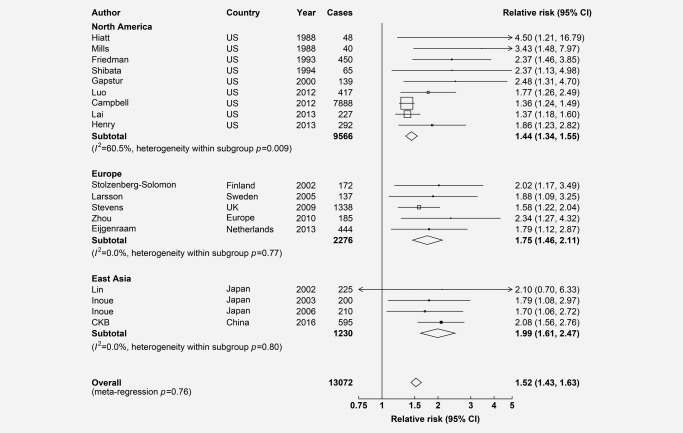
Adjusted RRs for PC associated with previously diagnosed diabetes in meta‐analysis of CKB and 17 published studies, by regions. Boxes represent the relative risks (RRs) associated with previously diagnosed diabetes for individual studies, with the size of the box inversely proportional to the variance of the logRR. Open boxes represent previously published studies and the black box represents CKB. Diamonds represent summary RRs for overall and each region. Within categories RRs are ordered according to their year of publication. Estimates and 95% CI of the summary RRs are in bold.

Among 18 studies including CKB, involving 4.1 million individuals with 13,072 cases of PC, previously diagnosed diabetes was associated with a 52% (RR = 1.52 [1.43–1.63]; *n* = 18) increased risk of PC, with moderate heterogeneity between studies (*I* ^2^ = 49%, Cochran's Q *p* = 0.01; Fig. 2). The RRs were greater in East Asian (1.99, 1.61–2.47; *n* = 4) and European studies (1.75, 1.46–2.11; *n* = 5) than in North American studies (1.44, 1.34–1.55; *n* = 9). There was no significant heterogeneity between studies conducted in East Asia and Europe, but a large between‐study heterogeneity in North America (*I* ^2^ = 61%, Cochran's *Q p* = 0.009; Fig. 2). There was evidence of publication bias in studies conducted in North America (Egger's test *p* < 0.001), but not in Europe or East Asia (Egger's test *p* = 0.42, Supporting Information Fig. 3).

Six other studies also assessed the associations of both previously diagnosed and screen‐detected diabetes (*n* = 5) or of screen‐detected (*n* = 1) diabetes with PC. When meta‐analysed together with CKB, there was little heterogeneity in risk estimates according to how diabetes was ascertained, although the ability to detect a difference was limited due to the small number of studies involved (Supporting Information Fig. 4). For previously diagnosed diabetes, studies with adjustment for BMI appeared to have lower RRs (1.46 [1.36–1.56] with adjustment vs. 2.11 [1.81–2.45] without adjustment for BMI, Supporting Information Fig. 5). Likewise, studies with exclusion of more years of follow‐up also tended to show somewhat lower RRs (Supporting Information Fig. 6). But, apart from CKB, few other studies had compared directly the RRs with exclusion of different years of follow‐up within the same study. In meta‐analysis, there was no significant trend for studies reporting longer duration of diabetes at study enrolment to have greater risk of PC (Supporting Information Fig. 7).

**Figure 3:**
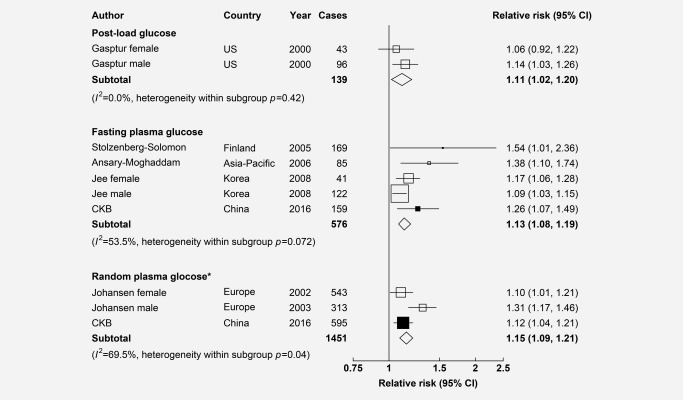
Adjusted RRs for PC associated with each 1 mmol/L increase in blood glucose in meta‐analysis of CKB and 5 published studies among those without previously diagnosed diabetes. Convention as in Figure 2. 1 mmol/L= 18mg/dL. * These studies adjusted for regression dilution.

In a meta‐analysis of CKB and five published studies, among participants without diabetes, each 1 mmol/L increase in blood glucose was associated with a 15% (RR = 1.15, 1.09–1.21) increased risk of PC for RPG, a 13% (1.13 [1.08–1.19]) for FPG and a 11% (1.11 [1.02–1.20]) for post‐load glucose (Fig. 3). There was little heterogeneity in risk estimates across different studies or by different blood glucose measures (meta‐regression *p* = 0.52).

## Discussion

This is the first large prospective study of the association between diabetes, blood glucose and risk of PC in China. We identified individuals with diabetes to have almost a twofold increased risk of PC and the risk also appeared to increase progressively with increasing duration of diabetes. Among those without a prior diagnosis of diabetes, there was also a strong positive dose‐response association of RPG with PC incidence. The results persisted after excluding the first 2 or 5 years of follow‐up. Our results are largely consistent with previous studies in Western and other East Asian populations, as indicated in the presented meta‐analyses.

Almost three dozen studies have previously assessed the associations of diabetes with risk of PC. The majority of these studies were from high‐income countries where the prevalence of diabetes was higher and the mean duration of diabetes was longer than CKB. Moreover, most studies focused primarily on previously diagnosed diabetes. Our risk estimate for previously diagnosed diabetes was consistent with studies in other East Asian and European populations, but more extreme than studies conducted in the US where the risk estimates varied significantly across studies for reasons that were not fully understood. Only six previous studies included information on screen‐detected diabetes^20^, ^21^, ^22^, ^23^, ^24^, ^25^ and the proportion of screen‐detected versus previously diagnosed diabetes was comparable with those in CKB, as was the overall proportional excess risk (1.87 vs. 1.82). Only one study presented separate results for screen‐detected diabetes,^25^ with a similar risk estimate as in CKB.

In our meta‐analysis, studies with adjustment for BMI appeared to have lower relative risks of PC with diabetes compared with those without adjustment. However, few previous studies presented simultaneously the risk estimates before and after adjustment for BMI so it is difficult to establish reliably whether the difference in risk estimates was due to difference in adjustment for adiposity or other factors. In CKB, adjustment for BMI and various other measures of adiposity had negligible effects on the risk estimates (Supporting Information Table 3), suggesting that adiposity played little role in mediating or confounding the association between diabetes and PC in Chinese populations. This finding appeared to be consistent with the limited evidence from other East Asian studies^26^ but contrasted with studies in Western populations,^27^ where the mean BMI was much higher than in East Asian populations.

In contrast with CKB findings, three previous meta‐analyses of prospective cohort studies^6^, ^7^, ^8^ have reported a negative association of duration of diabetes with PC. It is possible that the high risk of PC among individuals with a shorter duration of diabetes might be due to reverse causality (i.e. diabetes induced by preclinical pancreatic cancer).^28^, ^29^ To address these issues, previous studies excluded early periods of follow‐up, examined PC risk by diabetes duration since study enrolment, or used a time‐dependent approach to account for diabetes duration. However, given the range of approaches used, it was challenging to combine appropriately the data from different studies. In our meta‐analysis, we only included studies that reported PC risk by diabetes duration at study enrolment, and observed a weak positive association between diabetes duration and PC, which was consistent with our findings in CKB.

Both animal and epidemiological studies have suggested that new‐onset diabetes might be a manifestation of, rather than an aetiological factor for, PC.^30^ On the other hand, new‐onset diabetes might be associated with PC due to increased ascertainment (i.e. patients with newly diagnosed diabetes are under increased medical surveillance and thus might be more likely to be diagnosed with PC or vice versa).^23^, ^31^ However, our findings did not appear to support this hypothesis. In CKB, the positive association between diabetes and PC persisted even after excluding the first 5 years of follow‐up.

A recent meta‐analysis of nine prospective studies reported a 14% increase risk of PC per 0.56 mmol/L increase in blood glucose across the range of pre‐diabetes and diabetes. However, most studies used FBG and only one study used RBG.^9^ Our findings among individuals without prior diagnosis of diabetes were largely in agreement with risk estimates in this meta‐analysis (HR per 1 mmol/L increase in blood glucose 1.12 vs. 1.15). In our meta‐analysis, we found little statistical heterogeneity in risk estimates by different blood glucose measures. Our findings in CKB and the meta‐analysis suggest that the positive association between blood glucose and PC risk extended down to below the diabetic and pre‐diabetic range and that RBG appears to be equally informative compared with FBG, at least in large population studies.

Hyperglycaemia and insulin resistance have been hypothesized as the underlying mechanisms linking diabetes and PC.^7^, ^32^, ^33^ Hyperglycaemia might increase the risk of PC by providing more glucose to fuel tumour growth, thereby enhancing proliferation and invasion ability of PC cells.^30^ Insulin has growth promoting and mitogenic effects on PC cells.^32^, ^33^ Insulin also indirectly impacts pancreatic carcinogenesis by increasing bioavailability of insulin‐like growth factor (IGF), a multifunctional peptide that regulates cell proliferation, differentiation and apoptosis.^34^, ^35^

The strengths of CKB include a prospective design, a large and diverse study population (urban and rural), and careful adjustment for risk factors for PC. In addition, we examined separately screen‐detected diabetes, which represented undiagnosed diabetes not affected by treatment or lifestyle changes. One major limitation of CKB was that screen‐detected diabetes was defined using RPG, and thus may be subject to greater misclassification.^36^ However, our result was highly consistent with previous studies that used FBG, post‐load plasma glucose or HbA1C to define screen‐detected diabetes.

In summary, among Chinese adults, diabetes was associated with almost twofold increased risk of PC. Moreover, among participants without prior diagnosis of diabetes, RPG was associated positively with risk of PC. Our results suggest that diabetes might be an aetiological factor of PC in Chinese, largely consistent with evidence from previous studies in Western populations. Further research is needed to determine whether diabetes or high plasma glucose is a causal factor for PC. Given that the majority of diabetes cases are undiagnosed in China,^11^ early detection of screen‐detected diabetes could help identify a group of individuals at increased risk of PC.

## Supporting information

Supporting Information
