# Supplementary material for: Diabetes, plasma glucose and incidence of pancreatic cancer: A prospective study of 0.5 million Chinese adults and a meta‐analysis of 22 cohort studies
Source: Int J Cancer. 2017 Feb 8;140(8):1781–8. doi: 10.1002/ijc.30599 (PMC5396360; doi:10.1002/ijc.30599)

**Supplementary Material**

**Diabetes, plasma glucose and incidence of pancreatic cancer: a prospective study of 0.5 million Chinese adults and a meta-analysis of 22 cohort studies**

Table of contents

[Supplementary Data Text. Search strategies 2](#_Toc467872194)

[Supplementary Data Text. References of the articles included in the meta-analysis 3](#_Toc467872195)

[Supplementary Table 1. Adjusted RRs for PC by characteristics of study in meta-analysis of CKB and other studies 7](#_Toc467872196)

[Supplementary Table 2. Adjusted HRs for PC by diabetes and RPG at baseline in CKB 8](#_Toc467872197)

[Supplementary Table 3. Adjusted HRs for PC associated with previously diagnosed diabetes with further adjustment for different measures of adiposity in CKB 9](#_Toc467872198)

[Supplementary Table 4. Adjusted HRs for PC by diabetes in CKB 10](#_Toc467872199)

[Supplementary Table 5. Adjusted HRs for PC mortality by diabetes and RPG at baseline in CKB 11](#_Toc467872200)

[Supplementary Table 6. Characteristics of prospective studies of diabetes and PC included in the meta-analysis 12](#_Toc467872201)

[Supplementary Figure 1. PRISMA flow chart of literature search 19](#_Toc467872202)

[Supplementary Figure 2. Adjusted RRs for PC associated with diabetes by study design in meta-analysis of CKB and 33 published studies 20](#_Toc467872203)

[Supplementary Figure 3. Funnel plot of previously diagnosed diabetes and PC in meta-analysis of CKB and 17 published studies 21](#_Toc467872204)

[Supplementary Figure 4. Adjusted RRs for PC associated with diabetes according to diagnostic methods in meta-analysis of CKB and 22 published studies 22](#_Toc467872205)

[Supplementary Figure 5. Adjusted RRs for PC associated with previously diagnosed diabetes with and without adjustment for BMI in meta-analysis of CKB and 17 published studies 23](#_Toc467872206)

[Supplementary Figure 6. Adjusted RRs for PC associated with diabetes by different exclusions of follow-up periods in meta-analysis of CKB and 22 published studies 24](#_Toc467872207)

[Supplementary Figure 7. Adjusted RRs for PC associated with duration of diabetes since diagnosis in meta-analysis of CKB and 4 published studies 25](#_Toc467872208)

# **Supplementary Data Text. Search strategies**

***Search 1: PubMed, inception through March 15, 2016***

(pancreatic neoplasms[MeSH terms]) OR

(pancreatic neoplas*[tiab] OR pancreas neoplas*) OR

(pancreatic cancer*[tiab] OR pancreas cancer*) OR

(pancreatic carcin*[tiab] OR pancreas carcin*) OR

(pancreatic tumo*[tiab] OR pancreas tumo*) OR

(pancreatic metasta*[tiab] OR pancreas metasta*) OR

(pancreatic malign*[tiab] OR pancreas malign*) OR

(pancreatic adenocarcinoma*[tiab] OR pancreas adenocarcinoma*)

AND ("diabetes mellitus"[Mesh]) OR (diabetes mellitus[tiab]) OR (diabet*[tiab]) OR (NIDDM[tiab])

***Search 2: Embase, inception through March 15, 2016***

pancreatic neoplasms/ OR

(pancreatic neoplas* OR pancreatic cancer* OR pancreatic carcin* OR pancreatic tumo* OR pancreatic metasta* OR pancreatic malign* OR pancreatic adenocarcinoma*).ti,ab.)

AND (diabetes mellitus/ OR (diabetes mellitus).ti.ab. OR (diabet*)).ti.ab. OR (NIDDM) ).ti.ab.

# **Supplementary Data Text. References of the articles included in the meta-analysis**

***Prospective cohort studies (n=16):***

Mills PK, Beeson WL, Abbey DE, et al. Dietary habits and past medical history as related to fatal pancreas cancer risk among Adventists. *Cancer* 1988;61(12):2578-85

Balkau B, Barrett-Connor E, Eschwege E, et al. Diabetes and pancreatic carcinoma. *Diabete Metab* 1993;19(5):458-62

Shibata A, Mack TM, Paganini‐Hill A, et al. A prospective study of pancreatic cancer in the elderly. *Int J Cancer* 1994;58(1):46-49

Gapstur SM, Gann PH, Lowe W, et al. Abnormal glucose metabolism and pancreatic cancer mortality. *JAMA* 2000;283(19):2552-8

Stolzenberg-Solomon RZ, Pietinen P, Taylor PR, et al. A prospective study of medical conditions, anthropometry, physical activity, and pancreatic cancer in male smokers (Finland). *Cancer Causes Control* 2002;13(5):417-426

Lin Y, Tamakoshi A, Kawamura T, et al. Risk of pancreatic cancer in relation to alcohol drinking, coffee consumption and medical history: findings from the Japan collaborative cohort study for evaluation of cancer risk. *Int J Cancer* 2002;99(5):742-746

Batty GD, Shipley MJ, Marmot M, et al. Diabetes status and post-load plasma glucose concentration in relation to site-specific cancer mortality: findings from the original Whitehall study. *Cancer Causes Control* 2004;15(9):873-81

Jee SH, Ohrr H, Sull JW, et al. Fasting serum glucose level and cancer risk in Korean men and women. *JAMA* 2005;293(2):194-202

Larsson SC, Permert J, Hakansson N, et al. Overall obesity, abdominal adiposity, diabetes and cigarette smoking in relation to the risk of pancreatic cancer in two Swedish population-based cohorts*. Br J Cancer* 2005;93(11):1310-5

Inoue M, Iwasaki M, Otani T, et al. Diabetes mellitus and the risk of cancer: results from a large-scale population-based cohort study in Japan. *Arch J Med* 2006;166(17):1871-1877

Stevens RJ, Roddam AW, Spencer EA, et al. Factors associated with incident and fatal pancreatic cancer in a cohort of middle-aged women. *Int J Cancer* 2009;124(10):2400-5

Luo J, Chlebowski R, Liu S, et al. Diabetes mellitus as a risk factor for gastrointestinal cancers among postmenopausal women. *Cancer Causes Control* 2013;24(3):577-585

Campbell PT, Newton CC, Patel AV, et al. Diabetes and cause-specific mortality in a prospective cohort of one million US adults. *Diabetes Care* 2012;35(9):1835-1844

Lai GY, Park Y, Hartge P, et al. The association between self-reported diabetes and cancer incidence in the NIH-AARP Diet and Health Study. *J Clin Endocrinol Metab* 2013;98(3):497-502

Henry SA, Prizment AE, Anderson KE. Duration of diabetes and pancreatic cancer in a case-control study in the Midwest and the Iowa Women's Health Study (IWHS) cohort. *JOP* 2013;14(3):243-9

De Bruijn KM, Ruiter R, de Keyser CE, et al. Detection bias may be the main cause of increased cancer incidence among diabetics: Results from the Rotterdam Study. *Eur J Cancer* 2014;50(14):2449-2455

***Nested case-control studies (n=4):***

Hiatt RA, Klatsky AL, Armstrong MA. Pancreatic cancer, blood glucose and beverage consumption. *Int J Cancer* 1988;41(6):794-797

Friedman DG, Van den Eeden SK. Risk factors for pancreatic cancer: an exploratory study. *Int J Epidemiol* 1993;22(1):30-37

Inoue M, Tajima K, Takezaki T, et al. Epidemiology of pancreatic cancer in Japan: a nested case-control study from the Hospital-based Epidemiologic Research Program at Aichi Cancer Center (HERPACC). *Int J Epidemiol* 2003;32(2):257-62

Grote VA, Rohrmann S, Nieters A, et al. Diabetes mellitus, glycated haemoglobin and C-peptide levels in relation to pancreatic cancer risk: a study within the European Prospective Investigation into Cancer and Nutrition (EPIC) cohort. *Diabetologia* 2011;54(12):3037-46

***Case-cohort study (n=1):***

Eijgenraam P, Heinen MM, Verhage BA, et al. Diabetes type II, other medical conditions and pancreatic cancer risk: a prospective study in The Netherlands. *Br J Cancer* 2013;109(11):2924-32

***Pooled analysis of prospective cohort studies (n=1):***

Zhou XH, Qiao Q, Zethelius B, et al. Diabetes, prediabetes and cancer mortality. *Diabetologia* 2010; 53(9): 1867-1876

***Record linkage studies (n=11):***

Jamal MM, Yoon EJ, Vega KJ, et al. Diabetes mellitus as a risk factor for gastrointestinal cancer among American veterans. *World J Gastroenterol* 2009;15(42):5274-8

El-Serag HB, Engels EA, Landgren O, et al. Risk of hepatobiliary and pancreatic cancers after hepatitis C virus infection: A population-based study of U.S. veterans. *Hepatology* 2009;49(1):116-23

Ogunleye AA, Ogston SA, Morris AD, et al. A cohort study of the risk of cancer associated with type 2 diabetes. *Br J Cancer* 2009;101(7):1199-201

Chodick G, Heymann AD, Rosenmann L, et al. Diabetes and risk of incident cancer: a large population-based cohort study in Israel. *Cancer Causes Control* 2010;21(6):879-887

Chen HF, Chen P, Li CY. Risk of malignant neoplasm of the pancreas in relation to diabetes: a population-based study in Taiwan. *Diabetes Care* 2011;34(5):1177-9

Johnson JA, Bowker SL, Richardson K, et al. Time-varying incidence of cancer after the onset of type 2 diabetes: evidence of potential detection bias. *Diabetologia* 2011;54(9):2263-2271

Wotton CJ, Yeates DGR, Goldacre MJ. Cancer in patients admitted to hospital with diabetes mellitus aged 30 years and over: record linkage studies. *Diabetologia* 2011;54(3):527-534

Atchison EA, Gridley G, Carreon JD, et al. Risk of cancer in a large cohort of US veterans with diabetes. *Int J Cancer* 2011;128(3):635-643

Lu Y, Rodríguez LAG, Malgerud L, et al. New-onset type 2 diabetes, elevated HbA1c, anti-diabetic medications, and risk of pancreatic cancer. *Br J Cancer* 2015;113:1607-1614

Dankner R, Boffetta P, Balicer RD, et al. Time-dependent risk of cancer after a diabetes diagnosis in a cohort of 2.3 million adults. *Am J Epidemiol* 2016;183(12):1098-1106

***Self-report of physician diagnosis of diabetes (n=16):***

Hiatt RA, Klatsky AL, Armstrong MA. Pancreatic cancer, blood glucose and beverage consumption. *Int J Cancer* 1988;41(6):794-797

Mills PK, Beeson WL, Abbey DE, et al. Dietary habits and past medical history as related to fatal pancreas cancer risk among Adventists. Cancer 1988;61(12):2578-85

Friedman DG, Van den Eeden SK. Risk factors for pancreatic cancer: an exploratory study. *Int J Epidemiol* 1993;22(1):30-37

Shibata A, Mack TM, Paganini‐Hill A, et al. A prospective study of pancreatic cancer in the elderly. *Int J Cancer* 1994;58(1):46-49

Gapstur SM, Gann PH, Lowe W, et al. Abnormal glucose metabolism and pancreatic cancer mortality. *JAMA* 2000;283(19):2552-8

Lin Y, Tamakoshi A, Kawamura T, et al. Risk of pancreatic cancer in relation to alcohol drinking, coffee consumption and medical history: findings from the Japan collaborative cohort study for evaluation of cancer risk. *Int J Cancer* 2002;99(5):742-746

Stolzenberg-Solomon RZ, Pietinen P, Taylor PR, et al. A prospective study of medical conditions, anthropometry, physical activity, and pancreatic cancer in male smokers (Finland). *Cancer Causes Control* 2002;13(5):417-426

Inoue M, Tajima K, Takezaki T, et al. Epidemiology of pancreatic cancer in Japan: a nested case-control study from the Hospital-based Epidemiologic Research Program at Aichi Cancer Center (HERPACC). *Int J Epidemiol* 2003;32(2):257-62

Larsson SC, Permert J, Hakansson N, et al. Overall obesity, abdominal adiposity, diabetes and cigarette smoking in relation to the risk of pancreatic cancer in two Swedish population-based cohorts. *Br J Cancer* 2005;93(11):1310-5

Inoue M, Iwasaki M, Otani T, et al. Diabetes mellitus and the risk of cancer: results from a large-scale population-based cohort study in Japan. *Arch J Med* 2006;166(17):1871-1877

Stevens RJ, Roddam AW, Spencer EA, et al. Factors associated with incident and fatal pancreatic cancer in a cohort of middle-aged women. *Int J Cancer* 2009;124(10):2400-5

Luo J, Chlebowski R, Liu S, et al. Diabetes mellitus as a risk factor for gastrointestinal cancers among postmenopausal women. *Cancer Causes Control* 2013;24(3):577-585

Campbell PT, Newton CC, Patel AV, et al. Diabetes and cause-specific mortality in a prospective cohort of one million US adults. *Diabetes Care* 2012;35(9):1835-1844

Henry SA, Prizment AE, Anderson KE. Duration of diabetes and pancreatic cancer in a case-control study in the Midwest and the Iowa Women's Health Study (IWHS) cohort. *JOP* 2013;14(3):243-9

Lai GY, Park Y, Hartge P, et al. The association between self-reported diabetes and cancer incidence in the NIH-AARP Diet and Health Study. *J Clin Endocrinol Metab* 2013;98(3):497-502

Eijgenraam P, Heinen MM, Verhage BA, et al. Diabetes type II, other medical conditions and pancreatic cancer risk: a prospective study in The Netherlands. *Br J Cancer* 2013;109(11):2924-32

Henry SA, Prizment AE, Anderson KE. Duration of diabetes and pancreatic cancer in a case-control study in the Midwest and the Iowa Women's Health Study (IWHS) cohort. *JOP* 2013;14(3):243-9

***Combining both a self-report of physician diagnosis of diabetes and screen-detected diabetes (n=5):***

Balkau B, Barrett-Connor E, Eschwege E, et al. Diabetes and pancreatic carcinoma. *Diabete Metab* 1993;19(5):458-62

Batty GD, Shipley MJ, Marmot M, et al. Diabetes status and post-load plasma glucose concentration in relation to site-specific cancer mortality: findings from the original Whitehall study. *Cancer Causes Control* 2004;15(9):873-81

Jee SH, Ohrr H, Sull JW, et al. Fasting serum glucose level and cancer risk in Korean men and women. *JAMA* 2005;293(2):194-202

Grote VA, Rohrmann S, Nieters A, et al. Diabetes mellitus, glycated haemoglobin and C-peptide levels in relation to pancreatic cancer risk: a study within the European Prospective Investigation into Cancer and Nutrition (EPIC) cohort. *Diabetologia* 2011;54(12):3037-46

De Bruijn KM, Ruiter R, de Keyser CE, et al. Detection bias may be the main cause of increased cancer incidence among diabetics: Results from the Rotterdam Study. *Eur J Cancer* 2014;50(14):2449-2455

***Previously diagnosed and screen-detected diabetes reported separately (n=1):***

Zhou XH, Qiao Q, Zethelius B, et al. Diabetes, prediabetes and cancer mortality. *Diabetologia* 2010; 53(9): 1867-1876

***Studies with information on duration of diabetes (n=4):***

Jee SH, Ohrr H, Sull JW, et al. Fasting serum glucose level and cancer risk in Korean men and women. *JAMA* 2005;293(2):194-202

Luo J, Chlebowski R, Liu S, et al. Diabetes mellitus as a risk factor for gastrointestinal cancers among postmenopausal women. *Cancer Causes Control* 2013;24(3):577-585

Henry SA, Prizment AE, Anderson KE. Duration of diabetes and pancreatic cancer in a case-control study in the Midwest and the Iowa Women's Health Study (IWHS) cohort. *JOP* 2013;14(3):243-9

De Bruijn KM, Ruiter R, de Keyser CE, et al. Detection bias may be the main cause of increased cancer incidence among diabetics: Results from the Rotterdam Study. *Eur J Cancer* 2014;50(14):2449-2455

# **Supplementary Table 1. Adjusted RRs for PC by characteristics of study in meta-analysis of CKB and other studies**

|  |  | **Fixed effects** | **Random effects** | **I^2^ (%)** | **p_h_1** | **p_h_2** |
| --- | --- | --- | --- | --- | --- | --- |
| **Subgroup** | **n** | **RR (95% CI)** | **RR (95% CI)** |  |  |  |
| Overall | 34 | 1.98 (1.92, 2.03) | 2.01 (1.73, 2.33) | 94.7 | <0.001 |  |
| Study design |  |  |  |  |  | 0.68 |
| Cohort | 19 | 1.55 (1.46, 1.64) | 1.79 (1.58, 2.03) | 55.9 | 0.002 |  |
| Nested case-control | 4 | 2.01 (1.53, 2.62) | 2.01 (1.53, 2.62) | 0.0 | 0.48 |  |
| Record linkage | 11 | 2.12 (2.05, 2.19) | 2.11 (1.63, 2.72) | 98.0 | <0.001 |  |
| Diagnostic method |  |  |  |  |  | 0.69 |
| Previously diagnosed | 18 | 1.52 (1.43, 1.63) | 1.78 (1.57, 2.02) | 49.0 | 0.01 |  |
| Screen-detected | 2 | 1.59 (1.17, 2.17) | 1.59 (1.17, 2.17) | 0.0 | 0.84 |  |
| Combined | 6 | 1.82 (1.61, 2.06) | 1.91 (1.58, 2.30) | 37.2 | 0.15 |  |
| Region |  |  |  |  |  | 0.76 |
| North America | 9 | 1.44 (1.34, 1.55) | 1.74 (1.45, 2.09) | 60.5 | 0.009 |  |
| Europe | 5 | 1.75 (1.46, 2.11) | 1.75 (1.46, 2.11) | 0.0 | 0.77 |  |
| Asia | 4 | 1.99 (1.61, 2.47) | 1.99 (1.61, 2.47) | 0.0 | 0.8 |  |
| BMI adjustment |  |  |  |  |  | 0.014 |
| No | 11 | 2.11 (1.81, 2.45) | 2.11 (1.81, 2.45) | 0.0 | 0.91 |  |
| Yes | 9 | 1.46 (1.36, 1.56) | 1.59 (1.41, 1.81) | 48.7 | 0.05 |  |
| Exclusion of early periods |  |  |  |  |  | 0.91 |
| 0 years | 13 | 1.49 (1.40, 1.60) | 1.75 (1.55, 1.99) | 61.1 | 0.002 |  |
| 1 year | 5 | 2.11 (1.66, 2.67) | 2.11 (1.66, 2.67) | 0.0 | 0.73 |  |
| 2-4 years | 5 | 1.67 (1.40, 1.98) | 1.64 (1.40, 1.93) | 0.0 | 0.79 |  |
| 5 years | 6 | 1.34 (1.21, 1.48) | 1.69 (1.22, 2.34) | 57.1 | 0.04 |  |
| >10 years | 5 | 1.34 (1.19, 1.50) | 2.02 (1.41, 2.91) | 73.1 | 0.005 |  |
| Duration of diabetes up to study enrolment | | |  |  |  | 0.54 |
| 1-5 years | 3 | 1.98 (1.46, 2.67) | 1.98 (1.46, 2.67) | 0.0 | 0.92 |  |
| 5-10 years | 4 | 2.29 (1.75, 3.00) | 2.29 (1.75, 3.00) | 0.01 | 0.45 |  |
| >10 years | 5 | 2.26 (1.79, 2.86) | 2.26 (1.79, 2.86) | 0.0 | 0.7 |  |

n denotes the number of studies of risk estimates.

p_h_1 for heterogeneity within each subgroup. p_h_2 for heterogeneity between subgroups with meta-regression analysis.

# **Supplementary Table 2. Adjusted HRs for PC by diabetes and RPG at baseline in CKB**

|  | **No. events** | **No. people** | **Rate,**  **per 100,000** | **HR (95% CI)**^1^ |
| --- | --- | --- | --- | --- |
| **Diabetes status** |  |  |  |  |
| No diabetes | 499 | 480307 | 103.89 | Reference |
| Total diabetes | 86 | 30007 | 286.67 | 1.87 (1.48, 2.37) |
|  |  |  |  |  |
| **Diabetes status** |  |  |  |  |
| No diabetes | 499 | 480307 | 103.89 | Reference |
| Screen-detected | 30 | 14007 | 214.18 | 1.54 (1.06, 2.23) |
| Previously diagnosed | 56 | 16000 | 350.00 | 2.13 (1.60, 2.84) |
|  |  |  |  |  |
| **Diabetes status**^2^ |  |  |  |  |
| No diabetes | 491 | 472699 | 103.87 | Reference |
| Screen-detected | 30 | 14007 | 214.29 | 1.18 (0.77, 1.79) |
| All diagnosed | 64 | 23608 | 271.19 | 2.39 (1.85, 3.09) |
|  |  |  |  |  |
| **Duration of diabetes**^3^ |  |  |  |  |
| No diabetes | 499 | 480307 | 103.89 | Reference |
| Screen-detected | 30 | 14007 | 214.18 | 1.54 (1.06, 2.23) |
| Previously diagnosed  <5 years | 21 | 8341 | 311.71 | 2.11 (1.41, 3.14) |
| ≥5 years | 30 | 7659 | 391.70 | 2.16 (1.48, 3.16) |
| *P for trend* |  |  |  | *0.01* |
|  |  |  |  |  |
| **Random plasma glucose** (mmol/L)^4^ | |  |  |  |
| ≤5.5 | 207 | 242737 | 85.28 | Reference |
| >5.5-7.0 | 208 | 172701 | 120.44 | 1.11 (0.91, 1.35) |
| ≥7.0 | 97 | 70751 | 137.10 | 1.22 (0.95, 1.58) |
| per 1 mmol/L^5^ | 512 | 486189 | 105.31 | 1.12 (1.04, 1.21) |

^1^ Estimates were stratified by age at risk, sex, and region, and adjusted for age at baseline, education, smoking, and alcohol.

^2^ Diabetes status was analysed as a time-updated variable in the Cox model.

^3^ Diabetes duration data missing or implausible for 20 participants.

^4^ The analysis for random plasma glucose was conducted in participants without previously diagnosed diabetes and additionally for fasting time.

^4^ HR per 1 mmol/L in blood glucose was corrected for regression dilution. The regression dilution ratio is 0.45. 1 mmol/L= 18mg/dL.

# **Supplementary Table 3. Adjusted HRs for PC associated with previously diagnosed diabetes with further adjustment for different measures of adiposity in CKB**

|  | **Total diabetes** | **Previously diagnosed** | **Screen-detected** |
| --- | --- | --- | --- |
| **Adjustment** | **HR (95% CI)** | **HR (95% CI)** | **HR (95% CI)** |
| Basic adjustment^1^ | 1.87 (1.48, 2.37) | 2.08 (1.56, 2.76) | 1.57 (1.08, 2.27) |
| + BMI | 1.85 (1.46, 2.35) | 2.06 (1.55, 2.73) | 1.52 (1.05, 2.21) |
| + Waist circumference | 1.86 (1.46, 2.36) | 2.05 (1.54, 2.73) | 1.53 (1.06, 2.23) |
| + Hip circumference | 1.86 (1.47, 2.36) | 2.07 (1.56, 2.75) | 1.54 (1.06, 2.23) |
| + Waist to hip ratio | 1.88 (1.48, 2.39) | 2.07 (1.56, 2.76) | 1.57 (1.08, 2.28) |
| + Percent body fat | 1.88 (1.48, 2.38) | 2.08 (1.56, 2.76) | 1.54 (1.06, 2.24) |

^1^ Basic adjustment included stratification by age at risk, sex, and region, and adjustment for age at baseline, education, smoking, and alcohol.

# **Supplementary Table 4. Adjusted HRs for PC by diabetes in CKB**

|  | **No. events** | **No. people** | **Rate,** | **HR (95% CI)**^1^ |
| --- | --- | --- | --- | --- |
|  |  |  | **per 100,000** |  |
| **Excluding the first two years**^2^ | |  |  |  |
| No diabetes | 421 | 475501 | 88.5 | Reference |
| Total diabetes | 63 | 29216 | 215.7 | 1.64 (1.25, 2.16) |
| Previously diagnosed | 39 | 15508 | 251.6 | 1.72 (1.23, 2.41) |
| Screen-detected | 24 | 13708 | 175.2 | 1.50 (0.99, 2.28) |
|  |  |  |  |  |
| **Excluding the first five years**^3^ | |  |  |  |
| No diabetes | 222 | 475302 | 46.74 | Reference |
| Total diabetes | 41 | 29194 | 140.46 | 2.06 (1.46, 2.91) |
| Previously diagnosed | 25 | 15494 | 161.29 | 2.09 (1.36, 3.20) |
| Screen-detected | 16 | 13700 | 116.79 | 1.93 (1.16, 3.23) |

^1^ Estimates were stratified by age at risk, sex, and region, and adjusted for age at baseline, education, smoking, and alcohol.

^2^ PC cases that occurred during the first two years of follow-up were excluded.

^3^ PC cases that occurred during the first five years of follow-up were excluded.

# **Supplementary Table 5. Adjusted HRs for PC mortality by diabetes and RPG at baseline in CKB**

|  | **No. events** | **No. people** | **Rate,**  **per 100,000** | **HR (95% CI)**^1^ |
| --- | --- | --- | --- | --- |
| **Diabetes status** |  |  |  |  |
| No diabetes | 361 | 480307 | 75.16 | Reference |
| Total diabetes | 59 | 30007 | 196.62 | 1.77 (1.33, 2.35) |
|  |  |  |  |  |
| **Diabetes status** |  |  |  |  |
| No diabetes | 361 | 480307 | 75.16 | 1.00 (0.89, 1.13) |
| Screen-detected | 17 | 14007 | 121.37 | 1.20 (0.75, 1.93) |
| Previously diagnosed | 42 | 16000 | 262.50 | 2.22 (1.63, 3.02) |
|  |  |  |  |  |
| **Duration of diabetes**^2^ |  |  |  |  |
| No diabetes | 361 | 480307 | 75.16 | 1.00 (0.88, 1.13) |
| Screen-detected | 17 | 14007 | 121.37 | 1.20 (0.75, 1.93) |
| Previously diagnosed  <5 years | 19 | 8341 | 227.79 | 2.14 (1.37, 3.36) |
| ≥5 years | 23 | 7659 | 300.30 | 2.28 (1.50, 3.46) |
| *P for trend* |  |  |  | *0.03* |
|  |  |  |  |  |
| **Random plasma glucose** (mmol/L)^3^ | |  |  |  |
| ≤5.5 | 148 | 242737 | 60.98 | 1.00 (0.84, 1.19) |
| >5.5-7.0 | 152 | 172701 | 88.01 | 1.14 (0.98, 1.33) |
| ≥7.0 | 66 | 70751 | 93.28 | 1.18 (0.92, 1.51) |
| per 1 mmol/L^4^ | 366 | 486189 | 75.28 | 1.11 (1.00, 1.24) |

^1^ Estimates were stratified by age at risk, sex, and region, and adjusted for age at baseline, education, smoking, and alcohol.

^2^ Diabetes duration data missing or implausible for 20 participants.

^3^ The analysis for random plasma glucose was restricted to participants without previously diagnosed diabetes and adjusting for fasting time.

^4^ HR per 1 mmol/L in blood glucose was corrected for regression dilution. The regression dilution ratio is 0.45. 1 mmol/L= 18mg/dL

# **Supplementary Table 6. Characteristics of prospective studies of diabetes and PC included in the meta-analysis**

| **Author, publication year, country/ region** | **Study name** | **Study design** | **Follow-up period, median/mean** | **Study size, age, number of cases** | **Exclusion of first few years of follow-up** | **Ascertainm-ent of diabetes** | **Description of categories** | **RR (95% CI)** | **Adjustment for confounders** |
| --- | --- | --- | --- | --- | --- | --- | --- | --- | --- |
| Dankner, 2016, Israel | Clalit Health Servises | Record linkage | 2002-2012, 11 years | 2186196, mean age: 46.6, 3928 cases | NA | Physician diagnosis | Overall  <1 year  1-2 years  2-11 years | 3.17 (2.90, 3.48)  14.55 (13.42, 15.78)  4.83 (4.04, 5.76)  2.91 (2.62, 3.23) | Age, socioecomonic status, ethnic group |
| Lu, 2015, UK | Health Improvement Network UK | Record linkage | 1996-2010, 6.8 years | 529 cases and 5000 controls, age 20-79 years | NA | Physician diagnosis | Overall  <1 year  1-2 years  2-5 years  >5 years | 2.16 (1.72, 2.72)  3.43 (2.06, 5.72)  2.28 (1.31, 4.00)  1.20 (0.71, 2.01)  1.00 | Age, sex, smoking, alcohol, BMI, Townsend deprivation index , calendar year, GP visit 1 year before index date |
| De Brujin, 2014, Netherlands | Rotterdam Study | Prospective cohort | 1990-2011, 11 years | 10181, mean age 69 years: 84 cases | NA | Previously diagnosed and screen-detected (fasting) | Overall  3 mo-5 years  >5 years | 3.60 (1.98, 6.41)  2.30 (0.63, 8.30)  1.01 (0.29, 3.15) | Age, sex, smoking, alcohol, year of age, sex, smoking status, alcohol, BMI, year of inclusion in the study |
| Eijgenraam, 2013, Netherland | Netherlands Cohort Study | Case-cohort | 1986-2011, 16.3 years | 5000 subcohort members, mean age 61 years: 448 cases | 5 years | Previously diagnosed | 5-10 years  ≥10 years | 1.45 (0.78, 2.70) 2.56 (1.26, 5.18) | Age, sex, smoking, alcohol, BMI, education, family history of pancreatic cancer |
| Henry, 2013, US | Iowa Women’s Health Study | Nested case-control | 1986-2008, 17.9 years | 36084 women, mean age 62.4 (cases) 61.7 (non-cases), 292 cases | 2 years | Previously diagnosed | Overall  2-5 years  5-10 years  ≥10 years | 1.86 (1.23, 2.83)  1.70 (0.78, 3.67)  2.62 (1.48, 4.65) 2.10 (1.36, 3.24) | Age, smoking, education, BMI |

| **Author, publication year, country/ region** | **Study name** | **Study design** | **Follow-up period, median/mean** | **Study size, age, number of cases** | **Exclusion of first few years of follow-up** | **Ascertainm-ent of diabetes** | **Description of categories** | **RR (95% CI)** | **Adjustment for confounders** |
| --- | --- | --- | --- | --- | --- | --- | --- | --- | --- |
| Lai, 2013, USA | NIH-AARP | Prospective cohort | 1995-2006, 10 years | 494867, age 50-71 years: 227 cases | NA | Previously diagnosed | Female  Male | 1.15 (0.85, 1.54)  1.46 (1.22, 1.73) | Age, sex, smoking, BMI, race, education, marital status, family history of cancer, self-reported health status, intake of red meat, white meat, fruits, vegetables, alcohol, coffee, physical activity, multivitamin use |
| Campbell, 2012, USA | Cancer Prevention Study-II | Prospective cohort | 1982-2008, 26 years | 1053831, age ≥30 years: 7888 cases | 5 years | Previously diagnosed | Excld 5 years:  Female  Male  Excld 10 years:  Female  Male | 1.22 (1.04, 1.43) 1.33 (1.15, 1.54)  1.12 (0.93, 1.35)  1.28 (1.08, 1.53) | Age, sex, smoking, alcohol, obesity, chronic pancreatits, gallbladder disease |
| Luo, 2012, US | Women’s Health Initiative | Prospective cohort | 1993-2009, 10.3 years | 145765 women, age 50-79 years; 417 cases | 2 years | Previously diagnosed | <10 years  ≥10 years | 1.31 (0.82, 2.08)  2.18 (1.35, 3.50) | Age, alcohol, smoking, BMI, WHR, physical activity, diet/energy intake, race, education, HRT, NSAIDS |
| Chen, 2011, Taiwan | Medical Claim Data in Taiwan | Record linkage | 2000-2006 | 615532 diabetic patients and 614871controls, age >40 years: 1555 cases | 1 year | Physician diagnosis | Overall | 1.54 (1.39, 1.71) | Age, sex, geographic area, urbanization status, status of hepatitis B, hepatitis C, cholecystitis, cholangitis, cholelithiasis, choledocholithiasis, cholecystectomy, gastric ulcer, duodenal ulcer, gastrectomy |

| **Author, publication year, country/ region** | **Study name** | **Study design** | **Follow-up period, median/mean** | **Study size, age, number of cases** | **Exclusion of first few years of follow-up** | **Ascertainme-nt of diabetes** | **Description of categories** | **RR (95% CI)** | **Adjustment for confounders** |
| --- | --- | --- | --- | --- | --- | --- | --- | --- | --- |
| Atchison, 2011, US | United States Veterans Affairs | Record linkage | 1969-1996, 10.5 years (diabetics) 11.9 years (non-diabetics) | 4501578, age 18-100 years: 7639 cases | 1 year | Physician diagnosis | Overall  2-5 years  5-10 years  ≥10 years | 1.50 (1.42, 1.59)  1.83 (1.66, 2.02)  1.38 (1.23, 1.54)  1.33 (1.20, 1.47) | Age, time, latency, race , number of visits, diagnoses of alcohol-related conditions, obesity, COPD |
| Johnson, 2011, Canada | British Columbia Linked Health Databases | Record linkage | 1994-2006, 4.3 years (diabetics) 4.4 years (non-diabetics) | 185100 diabetics, 185100 controls, mean age 60.7 years: 883 cases | 2 years | Physician diagnosis | 3 mo-1year  1-2 years  2-3 years  3-10 years | 3.71 (2.55, 5.39)  2.94 (2.00, 4.33)  1.78 (1.14, 2.77)  1.65 (1.28, 2.13) | Age, sex, SES, frequency of physician visits in the 2 years prior to the index date and index year |
| Wotton, 2011, UK | Oxford Record Linkage Study | Record linkage | 1963-2008 | 23669, age >30 years: 3622 cases | 3 years | Physician diagnosis | ORLS1:  Overall  0-5 years  ≥5 years  ORLS2:  Overall  0-5 years  ≥5 years | 2.21 (1.81, 2.67)  3.75 (2.95, 4.73)  0.93 (0.59, 1.40)  4.00 (2.92, 5.40)  4.25 (3.00, 5.91)  1.38 (0.43, 3.42) | Sex, age, time period in single calendar years, district of residence |
| Grote, 2011, Europe | European Prospective Investigation into Cancer and Nutrition | Nested case-control | 1992-2006, 5.3 years | 466 cases and 466 controls, mean age 58 years | NA | Previously diagnosed and screen-detected (HbA1C) | Overall  >2 years | 1.74 (1.12, 2.71)  1.45 (0.89, 2.37) | Matching factors: centre, sex, age at recruitment, date at entry, time between blood sampling and last consumption of foods and drinks; adjusting variables: smoking and BMI |
| Chodick, 2010, Israel | Maccabi Healthcare Services registry | Record linkage | 1999-2008, 8 years | 100595, mean age: 61.6, 48 cases | NA | Physician diagnosis | Overall | 1.67 (1.81, 2.36) | Age, region, socioeconomic status, use of healthcare services a prior to index date, BMI, history of cardiovascular diseases |

| **Author, publication year, country/ region** | **Study name** | **Study design** | **Follow-up period, median/mean** | **Study size, age, number of cases** | **Exclusion of first few years of follow-up** | **Ascertainm-ent of diabetes** | **Description of categories** | **RR (95% CI)** | **Adjustment for confounders** |
| --- | --- | --- | --- | --- | --- | --- | --- | --- | --- |
| Zhou, 2010, Europe | Collaborative analysis of Diagnostic criteria in Europe Study | Pooled analysis of prospective cohort | 1966-2004, 15.8 years | 44655, age: 45-76, 185 cases | NA | Previously diagnosed and screen-detected (2h plasma glucose) | All diabetes  Known diabetes  Undiagnosed diabetes | 1.90 (1.24, 2.93)  2.34 (1.27, 4.32)  1.67 (0.97, 2.85) | Study cohort, age, sex, BMI, systolic blood pressure, cholesterol, smoking status |
| Ogunleye, 2009, UK | Scotland Healthcare Database Study | Record linkage | 1993-2004, 3.9 years | 9577 diabetics between and 19154 non-diabetics, mean age 62.8 years: 51 cases | 1 year | Physician diagnosis | Overall | 2.85 (1.27, 6.43) | Deprivation decile |
| Stevens, 2009, UK | Million Women Study | Prospective cohort | 1996-2007, 7.2 years | 1.3 million middle-aged women, mean age 55.7 years: 1338 cases | 2 years | Previously diagnosed | Excld 2 years  Excld 4 years | 1.51 (1.13, 2.03)  1.57 (1.12, 2.20) | Age, smoking, region, socioeconomic status, BMI, height |
| El-Serag, 2009, US | Veteran Affairs Medical Cohort | Record linkage | 1988-2004, 2.3 years | 718687, mean age 52 years: 617 cases | 6 months | Previously diagnosed and measured | Overall | 1.24 (1.03, 1.49) | Age, sex, baseline visit date, type of visit (inpatient or outpatient) for the baseline visit and a preceding visit |

| **Author, publication year, country/ region** | **Study name** | **Study design** | **Follow-up period, median/mean** | **Study size, age, number of cases** | **Exclusion of first few years of follow-up** | **Ascertainm-ent of diabetes** | **Description of categories** | **RR (95% CI)** | **Adjustment for confounders** |
| --- | --- | --- | --- | --- | --- | --- | --- | --- | --- |
| Jamel, 2009, US | Veteran Affairs Medical Study | Record linkage | 1990-2000 | 278761 patients with type 2 DM and 836283 controls, mean age 65.8 (cases) 64.8 (controls): 2509 cases | 3 years | Physician diagnosis | Overall | 3.22 (3.03, 3.42) | Age, smoking, ethnicity, BMI |
| Inoue, 2006, Japan | Japan Public Health Center-Based Prospective Study | Prospective cohort | 1999-2003, 10.7 years | 97771, mean age 51.5; 210 cases | 5 years | Previously diagnosed | Overall | 1.78 (1.00, 3.20) | Age, smoking, alcohol, study area, history of cerebrovascular disease, IHD, BMI, leisure-time physical activity, coffee intake, green vegetable intake |
| Jee, 2005, Korea | Korean Cancer Prevention Study | Prospective cohort | 1992-2002, 10 years | 1298385, age 30-95 years: 163 cases | 5 years | Previously diagnosed and screen-detected (fasting) | Female  Male  Male:  0-5 years  5-10 years  >10 years | 1.71 (1.25, 2.34)  1.71 (1.42, 2.06)  2.0 (1.2, 3.3)  2.4 (1.4, 4.3)  3.0 (1.8, 5.0) | Age, smoking, alcohol |
| Larsson, 2005, Sweden | Swedish Mammography Cohort and Cohort of Swedish Men | Prospective cohort | 1987-2004  follow-up: 6.6 years | 83053, mean age 60 (M) 62 (F): 136 cases | 1 year | Previously diagnosed | Overall | 1.97 (1.10, 3.53) | Age, smoking, alcohol, education, physical activity |

| **Author, publication year, country/ region** | **Study name** | **Study design** | **Follow-up period, median/mean** | **Study size, age, number of cases** | **Exclusion of first few years of follow-up** | **Ascertainme-nt of diabetes** | **Description of categories** | **RR (95% CI)** | **Adjustment for confounders** |
| --- | --- | --- | --- | --- | --- | --- | --- | --- | --- |
| Batty, 2004, UK | Whitehall Study | Prospective cohort | 1967-1995, 25 years | 18403 men, age 40-64 years: 114 cases | 10 years | Previously diagnosed and screen-detected (post-load) | Overall  Excld 10 years | 3.99 (1.44, 11.0)  3.34 (0.81, 13.8) | Age, smoking, employment grade, physical activity, SBP, blood pressure-lowering medication, marital status, disease at study entry, unexplained weight loss, and mediating variables (BMI, triceps skinfold thickness, height adjusted FEV1, plasma cholesterol) |
| Inoue, 2003, Japan | Hospital-based Epidemiologic Research Program at Aichi Cancer Center | Nested case-control | 1988-1999 | 2000 controls, mean age 60.5 (cases) 60.4 (controls);  200 cases | NA | Previously diagnosed | Overall | 1.79 (1.08, 2.97) | Age, sex, smoking, family history of pancreatic cancer, past/present history of diabetes, regular physical activity, bowel habits, raw vegetable intake, alcohol |
| Stolzenberg-Solomon, 2002, Finland | Alpha-Tocopherol,Beta-Carotene Cancer Prevention (ATBC) Study | Prospective cohort | 1985-1997, 10.2 years | 29048 male smokers,, age: 50-69 years; 172 cases | 5 years | Previously diagnosed | Overall | 2.23 (1.08, 4.60) | Age, smoking, activity, asthma, BP |
| Lin, 2002, Japan | Japan Collaborative Cohort Study for Evaluation of Cancer Risk | Prospective cohort | 1988-1997, 8.1 years | 99527, mean age 57.3 years; 225 cases | 1 year | Previously diagnosed | Overall | 2.10 (1.20, 10.90) | Age, sex, smoking |

| **Author, publication year, country/ region** | **Study name** | **Study design** | **Follow-up period, median/mean** | **Study size, age, number of cases** | **Exclusion of first few years of follow-up** | **Ascertainm-ent of diabetes** | **Description of categories** | **RR (95% CI)** | **Adjustment for confounders** |
| --- | --- | --- | --- | --- | --- | --- | --- | --- | --- |
| Gapstur, 2000, US | Chicago Heart Association Detection Project | Prospective cohort | 1963-1995, 25 years | 35658, age 15-90 years: 139 cases | NA | Previously diagnosed | Men  (Women: no case with DM) | 2.48 (1.25, 4.49) | Age |
| Shibata, 1994, US | Leisure World Cohort Study | Prospective cohort | 1981-1990, 7.2 years | 13979, mean age 75 years; 65 cases | NA | Previously diagnosed | ≤4 years  >4 years | 3.63 (1.22, 10.80)  1.76 (0.62, 4.96) | Age, sex, smoking |
| Friedman, 1993, US | Multiphase Check-up Study | Nested case-control | 1964-1988, 12 years | 779 cases 2687 controls, age 15-94 years; 450 cases | 1 year | Previously diagnosed | >1 year  >2 years | 2.37 (1.46, 3.85)  2.15(1.26, 3.67) | Age, weight |
| Balkau, 1993, France | Paris Government Agency Cohort | Prospective cohort | 1968-1988, 17 years | 6988 men, age 44-55 years, 312 cases | 5 years | Previously diagnosed and screen-detected DM (post-load) | Overall | 4.9 (1.3, 18) | Age, smoking |
| Hiatt, 1988, US | Kaiser Permanente Medical Care Program | Nested case-control | 1978-1984, 6 years | 122894, mean age: 40.8, 48 cases | 1 year | Previously diagnosed | Overall | 7.6 (1.4, 41.4) | Age, sex, race, smoking, alcohol, coffee |
| Mills, 1988, USA | Adventist Health Study | Prospective cohort | 1973-1984, 5.7 years | 34198, age: >25 years; 40 cases | 1 year | Previously diagnosed | Overall | 3.43 (1.47, 7.94) | Age, sex |

F, female; M, male; NA, data not applicable.

# **Supplementary Figure 1. PRISMA flow chart of literature search**

1970 duplicates removed

76 publications were assessed based on full-text and their references were reviewed for additional publications

4176 publications excluded not meeting inclusion criteria:

- non-human studies: 990
- did not report on the associations of interest: 903
- non-original articles: 2039
- not prospective study design: 92
- non-English articles: 100
- duplicate: 51
- cannot be found: 1

44 publications excluded:

- duplicate publications: 9
- did not provide risk estimates on BMI and PC: 25
- study type other than prospective study: 10

7 found in bibliographies

32 publications, corresponding to 33 individual studies, included for diabetes

6215 publications identified on initial search:

- 3069 Pubmed
- 3146 Embase

4245 abstracts screened based on title and abstract

# **Supplementary Figure 2. Adjusted RRs for PC associated with diabetes by study design in meta-analysis of CKB and 33 published studies**

**
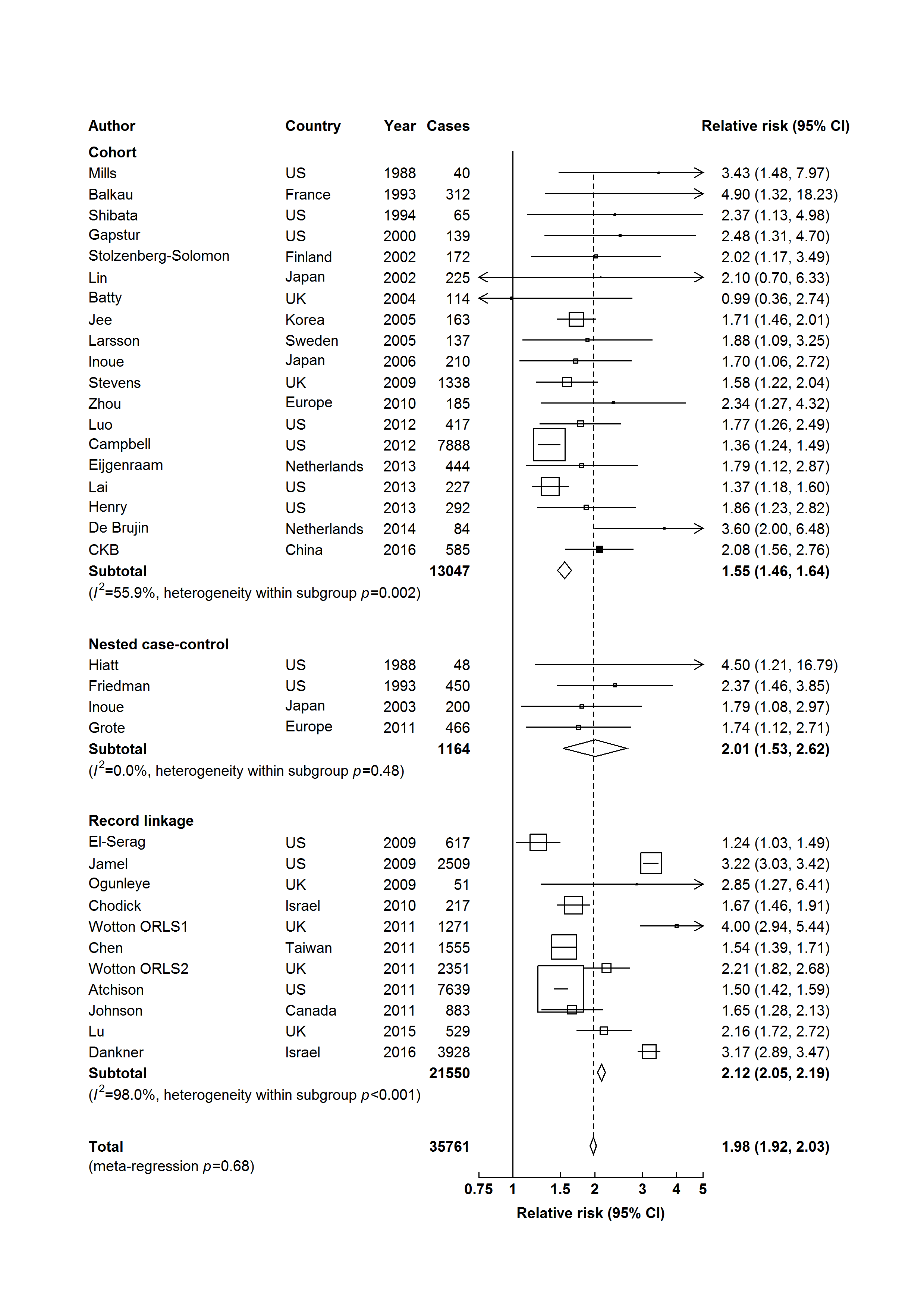
**

# **Supplementary Figure 3. Funnel plot of previously diagnosed diabetes and PC in meta-analysis of CKB and 17 published studies**

**
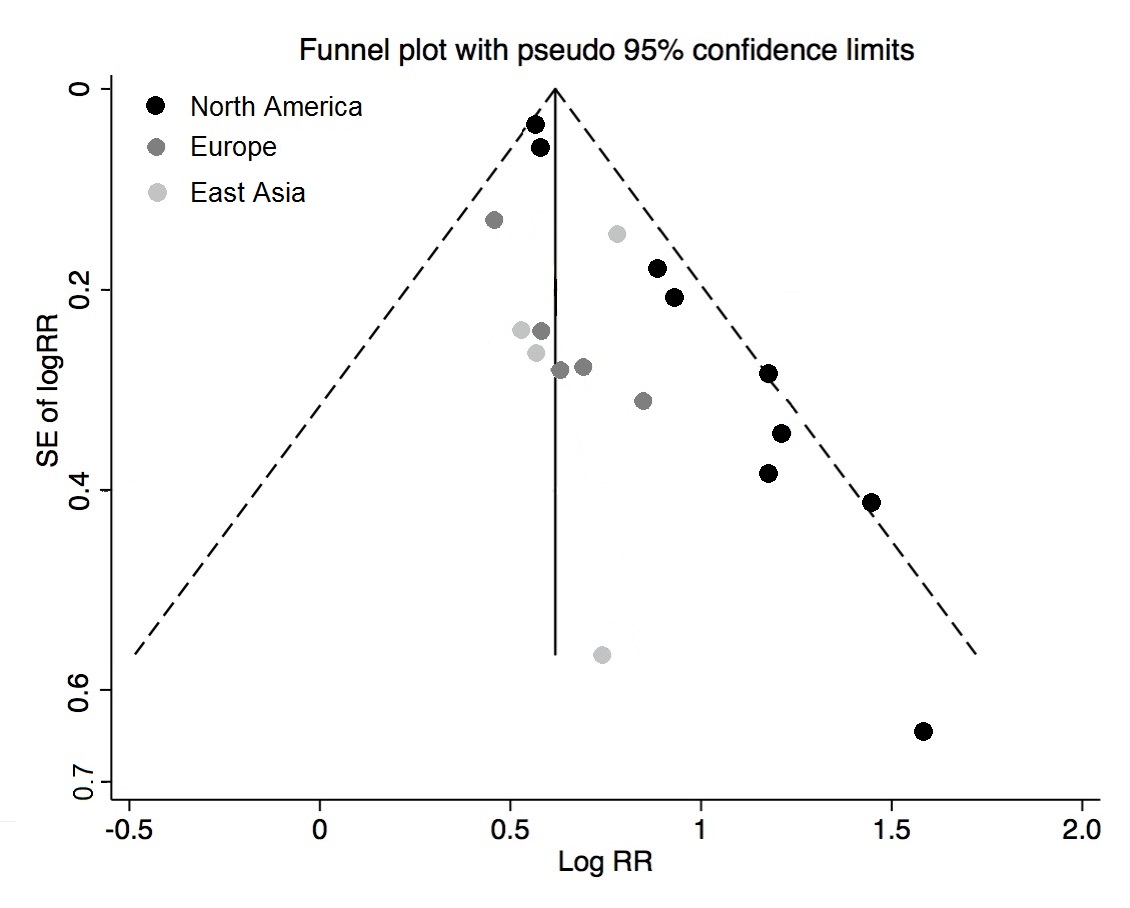
**

# **Supplementary Figure 4. Adjusted RRs for PC associated with diabetes according to diagnostic methods in meta-analysis of CKB and 22 published studies**

#


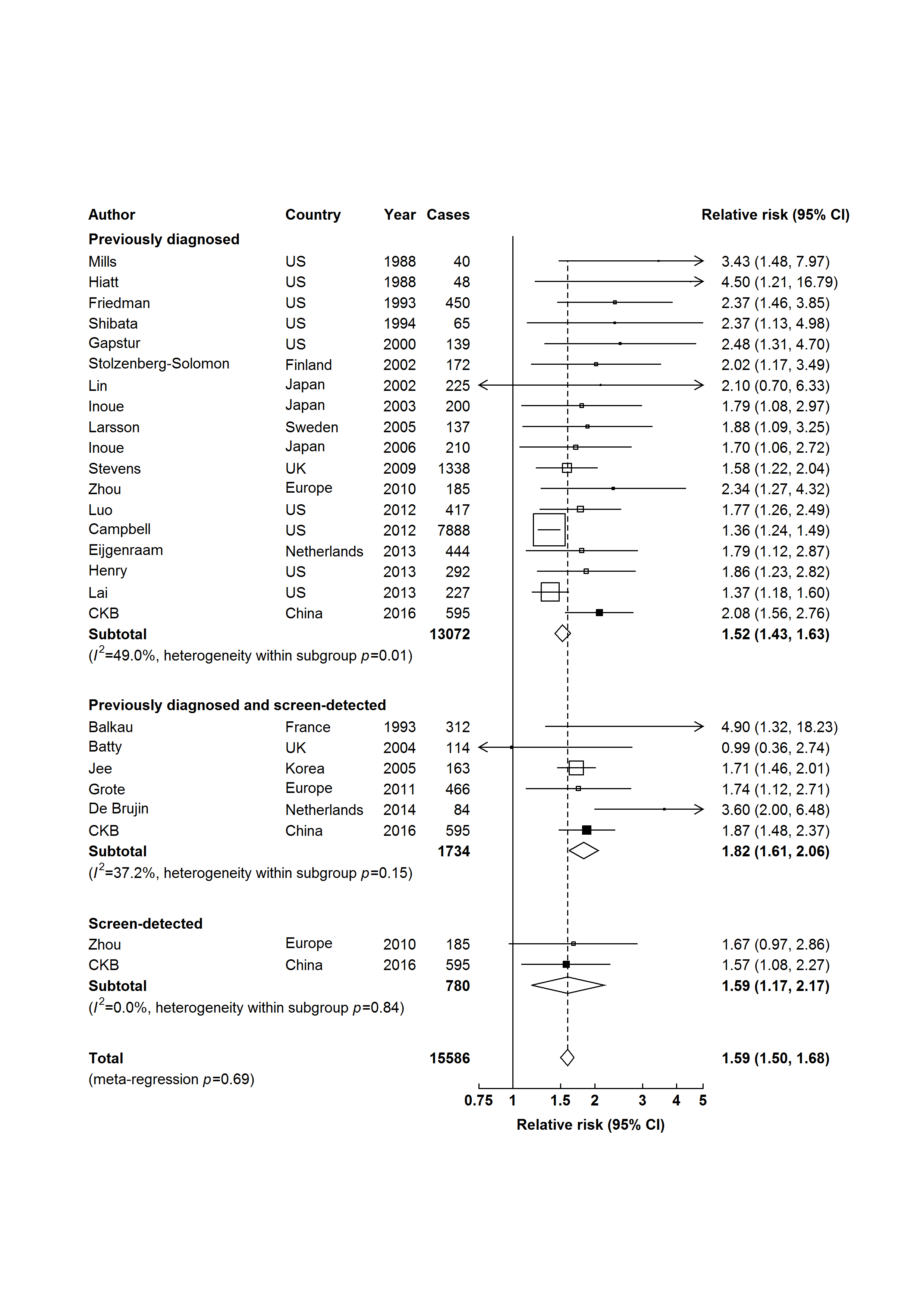


# **Supplementary Figure 5. Adjusted RRs for PC associated with previously diagnosed diabetes with and without adjustment for BMI in meta-analysis of CKB and 17 published studies**


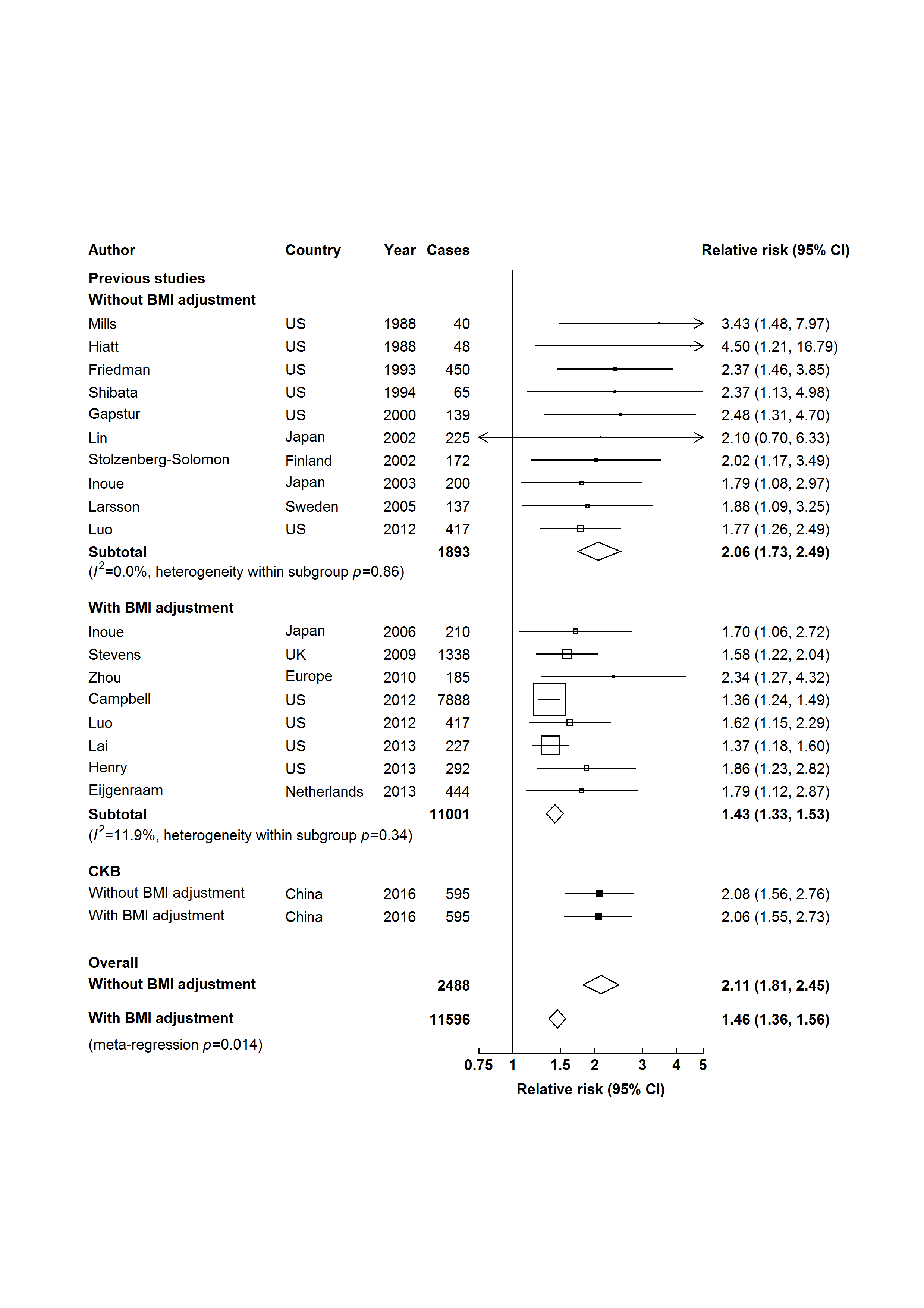


# **Supplementary Figure 6. Adjusted RRs for PC associated with diabetes by different exclusions of follow-up periods in meta-analysis of CKB and 22 published studies**

**
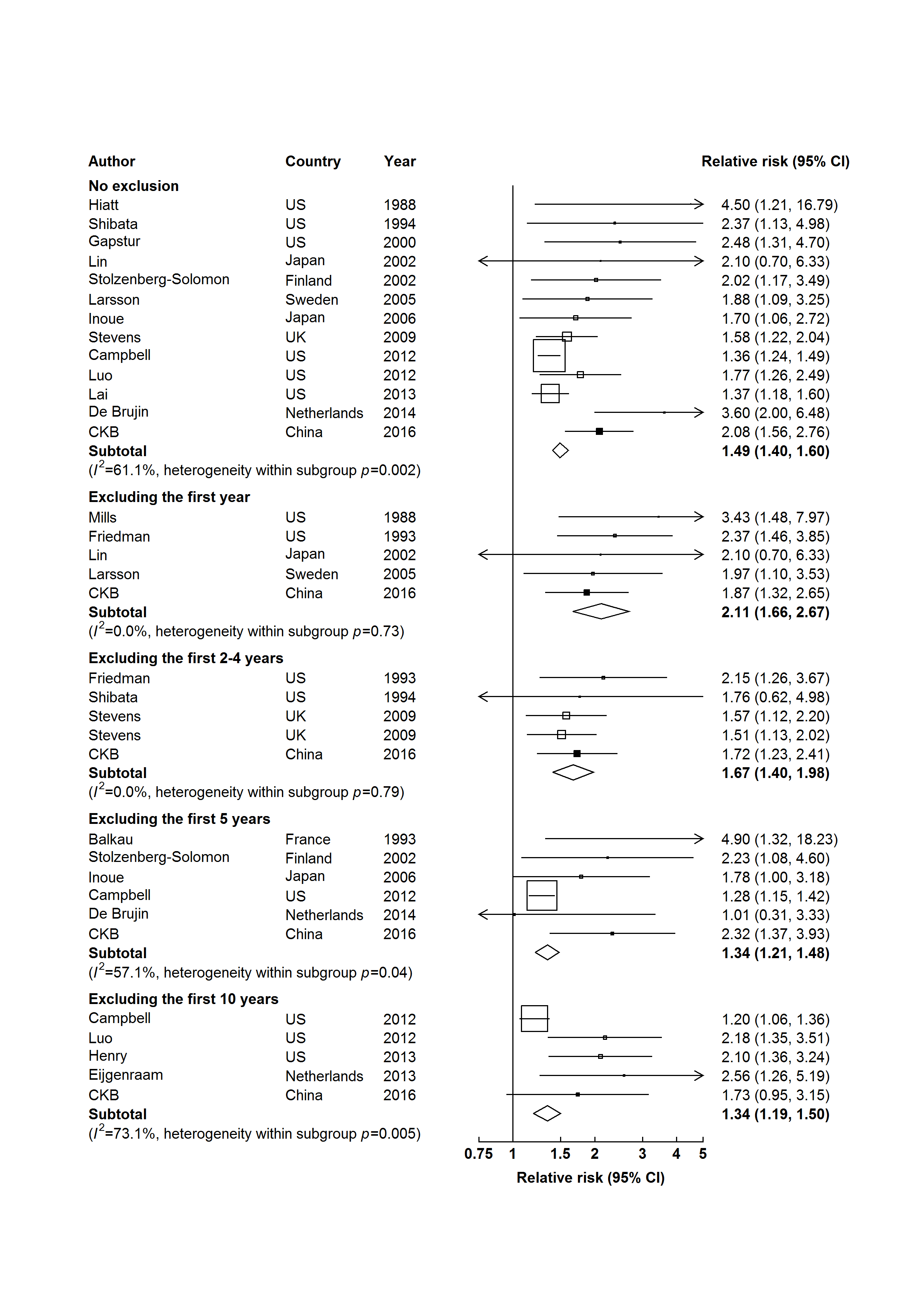
**

**Supplementary Figure 7. Adjusted RRs for PC associated with duration of diabetes since diagnosis in meta-analysis of CKB and 4 published studies**


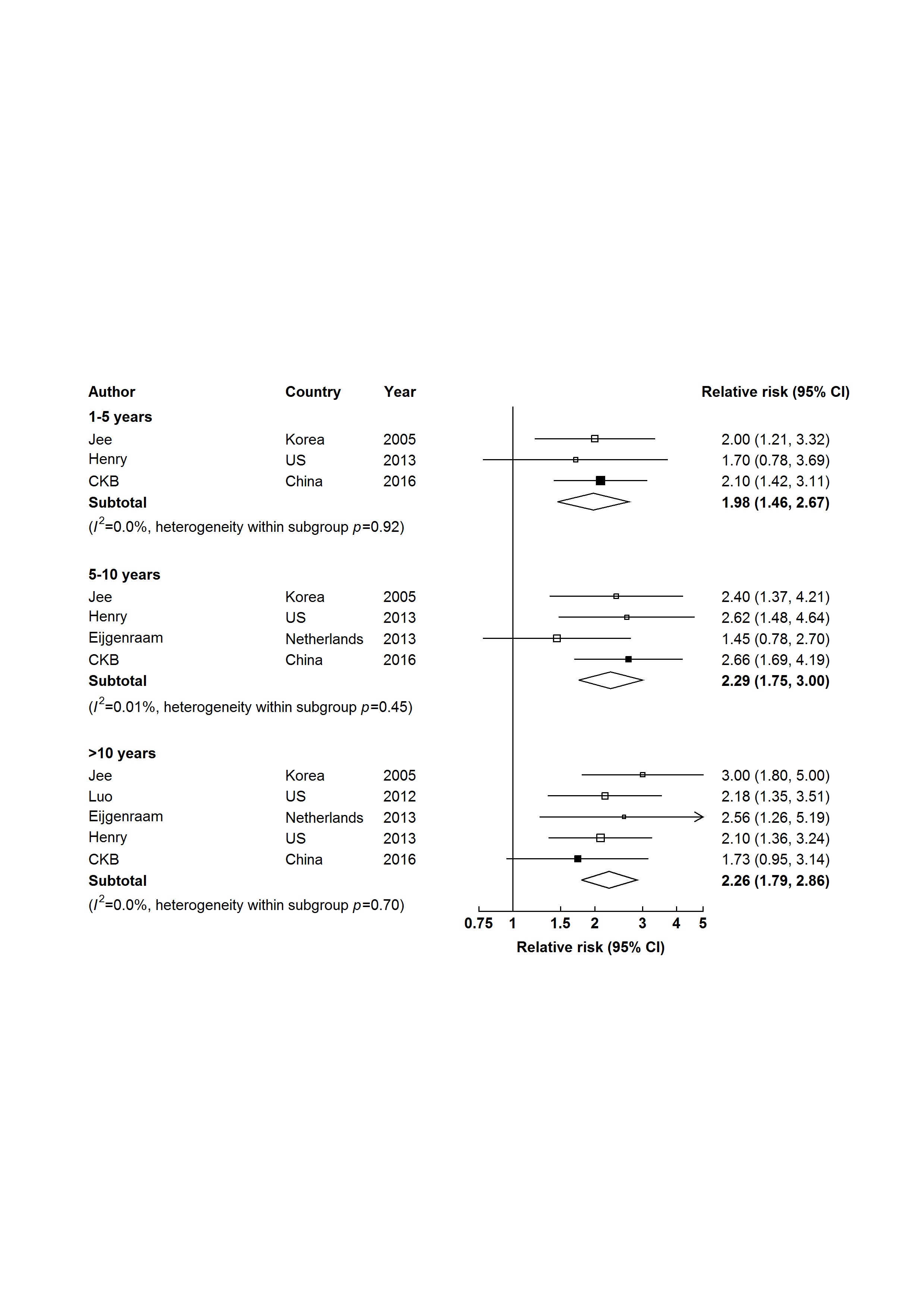

Supplement: Supplementary file 1 — Supporting Information [file IJC-140-1781-s001.docx]
